# Anthrax outbreak linked to consumption and handling of meat from unexpectedly deceased cattle, Kyotera district, Uganda, June–December 2023

**DOI:** 10.1186/s42522-025-00151-x

**Published:** 2025-05-07

**Authors:** Lawrence Tumusiime, Dominic Kizza, Anthony Kiyimba, Esther Nabatta, Susan Waako, Aggrey Byaruhanga, Benon Kwesiga, Richard Migisha, Lilian Bulage, Alex Riolexus Ario

**Affiliations:** 1 Uganda Public Health Fellowship Program, Uganda National Institute of Public Health, Kampala, Uganda; 2 Department of Integrated Epidemiology, Surveillance and Public Health Emergencies, Ministry of Health, Kampala, Uganda

**Keywords:** Anthrax, Cutaneous, Gastrointestinal, Outbreak, Uganda

## Abstract

**Background:**

Anthrax is an infectious zoonotic disease caused by gram-positive, rod-shaped, and spore-forming bacteria known as *Bacillus anthracis*. It continues to be a disease of public health importance in Uganda, with sporadic outbreaks reported annually in many parts of the country. In November 2023, Kyotera District reported a strange illness, characterized by itching, rash, swelling, and skin lesions which was later confirmed as anthrax. We investigated to assess its magnitude, identify potential exposures, and propose evidence-based control measures.

**Methods:**

A suspected cutaneous anthrax case was an acute onset of skin itching/swelling plus ≥ 2 of: skin reddening, lymphadenopathy, headache, fever or general body weakness. A suspected gastrointestinal anthrax case was an acute onset of ≥ 2 of: abdominal pain, vomiting, diarrhea, mouth lesions or neck swelling. A confirmed anthrax case was a suspected case with *Bacillus anthracis* PCR-positive results. To identify cases, we reviewed medical records and conducted community active case-finding. We conducted an unmatched case-control study and used logistic regression to identify risk factors of anthrax transmission. Controls were selected at a 1:4 ratio from the same villages as the case-patients.

**Results:**

We identified 63 cases (46 suspected and 17 confirmed); 48 (76%) were male. Of the 63, 55 cases (87%) were cutaneous and 8 (13%) were gastrointestinal, with a mean age of 42 years. Overall attack rate (AR) was 3.1/1,000; males were more affected (AR = 4.5/1,000) than females (AR = 1.5/1,000). Case-fatality rate was 19% (*n* = 12). Among the 63 cases, 18 (29%) sought care from health facilities; 33 (52%) were managed by traditional healers. The odds of anthrax infection were highest in individuals who both consumed and handled infected meat (OR = 20.9, 95% CI: 8.8–49.8), followed by those who only consumed the meat (OR = 5.81, 95% CI: 2.12–15.9).

**Conclusion:**

The anthrax outbreak in Kyotera District was primarily attributed to the consumption and handling of meat from cattle that had suddenly died. Poor health-seeking behavior and seeking care from traditional healers likely contributed to the high case fatality rate. To prevent future outbreaks, authorities should enforce cattle inspection protocols, expand anthrax vaccination campaigns, and enhance community education on safe meat handling and medical care-seeking practices.

## Background

Anthrax is an infectious zoonotic disease caused by gram-positive, rod-shaped, and spore-forming bacteria known as *Bacillus anthracis* [1]. Domestic and wild animals become infected when they inhale or ingest spores in contaminated soil, plants or water [2]. There are no reports of person-to-person transmission of anthrax; people get sick with anthrax through contact with infected animals or occupational exposure to infected or contaminated animal products (e.g. hair, wool, hides, bones) [3]. Human anthrax infection has four forms depending on the route of exposure; cutaneous, inhalational, gastrointestinal, and injectional [4]. Cutaneous anthrax is the most common form, accounting for > 95% of human cases, and results from direct contact with or exposure to infected animals or contaminated animal products [5].

Anthrax is a common disease with approximately 2,000 to 20,000 annual cases of human anthrax reported globally, with 75% occurring in African countries with low livestock vaccination rates [6]. Uganda has reported sporadic anthrax outbreaks in different sub-regions of the country [7]. The outbreaks mainly occur where people commonly keep livestock primarily within western, eastern, and northern Uganda [8–10].

On November 21, 2023, a video clip showing a strange illness in Kyotera District started circulating in newspapers, television, WhatsApp and several social media platforms with a local woman leader expressing the frustration of the local community for delayed feedback from the samples earlier taken by District Health team yet more deaths were happening. This illness was characterized by itching, rash, swelling, and skin lesions and was reported in Kabira Sub-County, Kyotera District. The report indicated that 35 cases, including 12 deaths, had occurred in Bwamiija and Ndolo parishes over two months. Subsequent investigations confirmed the illness as anthrax. With the high number of cases and deaths reported within a short period and the poor health-seeking behavior in this area, we investigated to determine what was the magnitude, and potential exposures, and propose evidence-based control measures.

## Methods

### Outbreak area

**Fig. 1:**
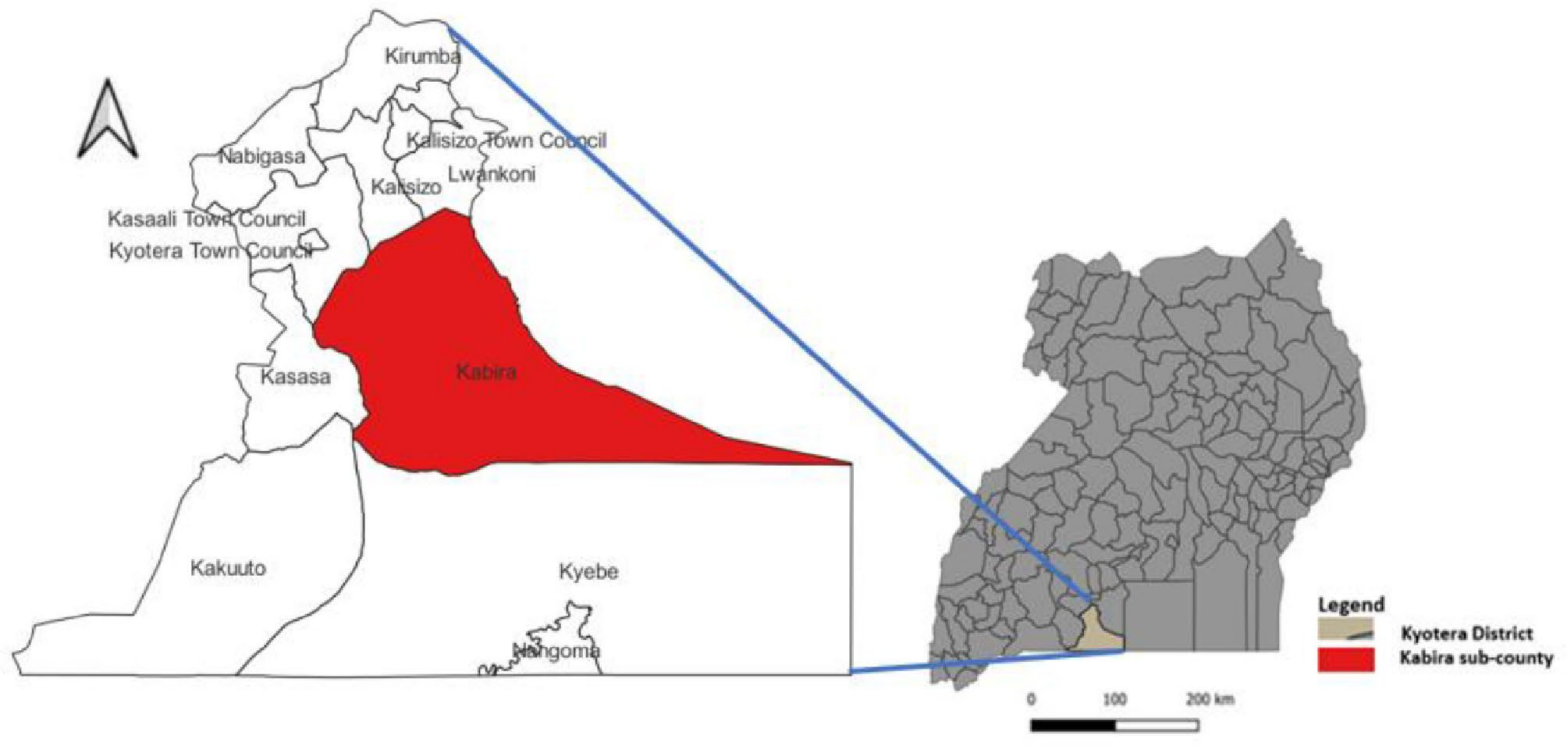
Location of Kyotera District in Uganda

The outbreak occurred in Kabira Sub-County, Kyotera District, situated in the cattle corridor in the southwestern part of Uganda (Fig. 1). The district covers a total area of 1,722 square kilometers and has a population of approximately 261,000 [11]. Agriculture is the backbone of the district’s economy, though most of it is at the subsistence level [12]. The majority of households (63%) have at least two cattle, contributing to an estimated cattle population of 68,893 [13]. The district is served by one public general hospital, with Kabira Sub-County served mainly by one public health facilities, one Private not for profit Health Centre IV, and numerous private clinics [14].

### Case definition and case finding

We defined a case of cutaneous anthrax as acute onset of skin itching or swelling plus ≥ 2 of skin reddening, lymphadenopathy, headache, fever, general body weakness in a resident of Kabira sub-county from June–December 2023. We defined a case of gastrointestinal anthrax as acute onset of ≥ 2 of the following symptoms: abdominal pain, vomiting, diarrhea, mouth lesions, neck swelling, in a resident of Kabira sub-county from June–December 2023. A Confirmed anthrax case was a suspected case with PCR-positive results for *Bacillus anthracis.*

We conducted house to house and health facility active case search using the case definition to identify case-patients in the affected areas from June 1–December 31, 2023. At the health facilities, we reviewed patient medical records to look for those consistent with our case definition. The community active case search was primarily done by visiting households in the villages with the guidance of the village health teams. The village health team members helped our team in mapping the households in their respective villages and then we systematically visited house to house to find cases. We also used snowballing to supplement on house to house case search by asking already identified case-patients to lead us to persons with similar signs and symptoms in the community.

### Descriptive epidemiology

We calculated proportions to describe the distribution of case-patients by age, sex, and symptoms. We also described case-patients by time of onset of symptoms using an epidemiological curve and calculated attack rates to describe the distribution of cases by age, sex, and parish of residence. We obtained denominator population to calculate the attack rates from the Uganda Bureau of Statistics data for Kyotera District.

### Laboratory investigations

Whole blood and swabs samples were collected from anthrax-suspected cases and available fluids from lesions. All samples were packaged using a triple package technique and transported using the hub system to the Uganda Virus Research Institute (UVRI) in Arua, Central Public Health Laboratories (CPHL). Total nucleic acid extraction was done using the Nuclisens EasyMag followed by Microbiome enrichment using the NEBNext Microbiome DNA Enrichment kit. Library preparation was done using the Illumina DNA Prep kit followed by genomic sequencing using the Illumina MiSeq next generation sequencing (NGS) platform. Data analysis was done using an in-house bioinformatics pipeline for metagenomics analysis as well as the Edge Bioinformatics pipeline. Bacillus Anthracis was confirmed by Kraken and Centrifuge metagenomics classification tools in the samples.

### Environmental investigations

We inspected animal farms in the affected villages using snowballing and house-to-house visits to identify farms that had reported sudden death of cattle, goats or sheep within Kabira Sub-county between June 1, 2023 and December 15, 2023. We interviewed the farm owner and herdsman to gather information on the farm management practices and how the meat and other animal products were distributed. We visited the affected farms and observed the pasture in the grazing area, and also areas where the cattle were slaughtered or buried. We interviewed the identified dealers of meat from cattle that had died suddenly and slaughtered for distribution to obtain information regarding where the meat was sold.

### Hypothesis generation interviews

We conducted interviews with the suspected case patients to identify possible sources and factors associated with contracting anthrax. We explored meat consumption from cattle that died suddenly, contact with livestock that suddenly died, the presence of skins and hides, animal ownership, and occupation.

### Case-control study

To test the hypothesis, we conducted a neighbourhoodmatched case-control study in Kabira sub-county, Kyotera District. We chose neighbourhood matching to control for potential confounding variables such as socioeconomic status, environmental factors, and access to healthcare [15]. We recruited and interviewed 50 out of the 63 case-patients on the line list because we could not get sufficient information on the 13 case-patients who had died and their relatives could not provide us with the information. For each case-patient, we selected 4 controls. A control was an individual who never had any signs of cutaneous or gastrointestinal anthrax from June 1, 2023 to the time of the investigation, resident in the same village as the case-patient. To randomly select control-persons, we obtained the locations of case-patients’ household and spun a randomizer bottle while at these households to obtain the first control household which was the nearest household in the direction shown by the bottle top. We spun the randomizer bottle to reduce selection bias and increase the validity of the study by ensuring that controls are similar to cases in terms of demographic and environmental characteristics [16]. All members present in the households at the time of data collection were listed and one was chosen randomly as a control. The randomizer bottle was spun after every interview at the control household for the next one until the four different households were obtained.

For each case and control, we obtained information on their meat consumption history, contact with dead livestock (slaughtering, dissecting, carrying), eating meat of an animal that had died suddenly, the clinical characteristics, as well as demographic variables.

### Data management and statistical analysis

After the data collection exercise, all the electronic questionnaires were saved as drafts in the kobo collect tool by the data collection assistants, checked for completeness, and the finalized forms were submitted. Appropriate codes were accorded to each question and checked through, per variable (column), to ensure that no double codes were entered and that no omissions had been made. Once the data management process was completed, then the data analysis process started using Stata version14, commencing with the analysis of frequency distributions, for all variables, yielding valid percentages. This was then followed by the conduction of bivariable analysis, in which each of the independent variables was analysed against the dependent variable.

We used logistic regression to identify factors associated with anthrax. Variables that had a p-value < 0.2 at bivariate level were included in the final model for multivariable analysis and corresponding adjusted odds ratios (aORs) and 95% confidence intervals were reported. Based on the findings from the multivariate analysis, we conducted a common reference group analysis between those who had consumed dead cattle meat and those with contact of suddenly dead cattle to unmask the true effects of the individual exposures [17].

### Ethical considerations

We conducted this activity in response to a public health emergency. The Ugandan MoH authorized us to conduct the investigation. This activity was also reviewed by the US CDC and was conducted consistent with applicable federal law and CDC policy. ^§ §^See e.g., 45 C.F.R. part 46, 21 C.F.R. part 56; 42 U.S.C. § 241(d); 5 U.S.C. § 552a; 44 U.S.C. § 3501 et seq. The office of the Center for Global Health, US Center for Disease Control and Prevention determined that this activity was not human subject research and with its primary intent being for public health practice or disease control.

We obtained administrative clearance conduct the activity from Kyotera District Local Government. We also sought administrative clearance from the respective local council one (LC 1) authorities where the outbreak occurred. LC 1 is the smallest administrative unit at the village level.

We obtained written informed consent from all the respondents who took part in the activity. They indicated their consent by accepting the team to tick an appropriate box in kobo collect for consent before proceeding with the interviews. Participants were assured that their participation was voluntary and that there would be no negative consequences for declining or withdrawing from the activity. Data collected did not contain any individual personal identifiers and information was stored in password-protected computers, which were inaccessible by anyone outside the investigation.

## Results

### Descriptive epidemiology

**Fig. 2:**
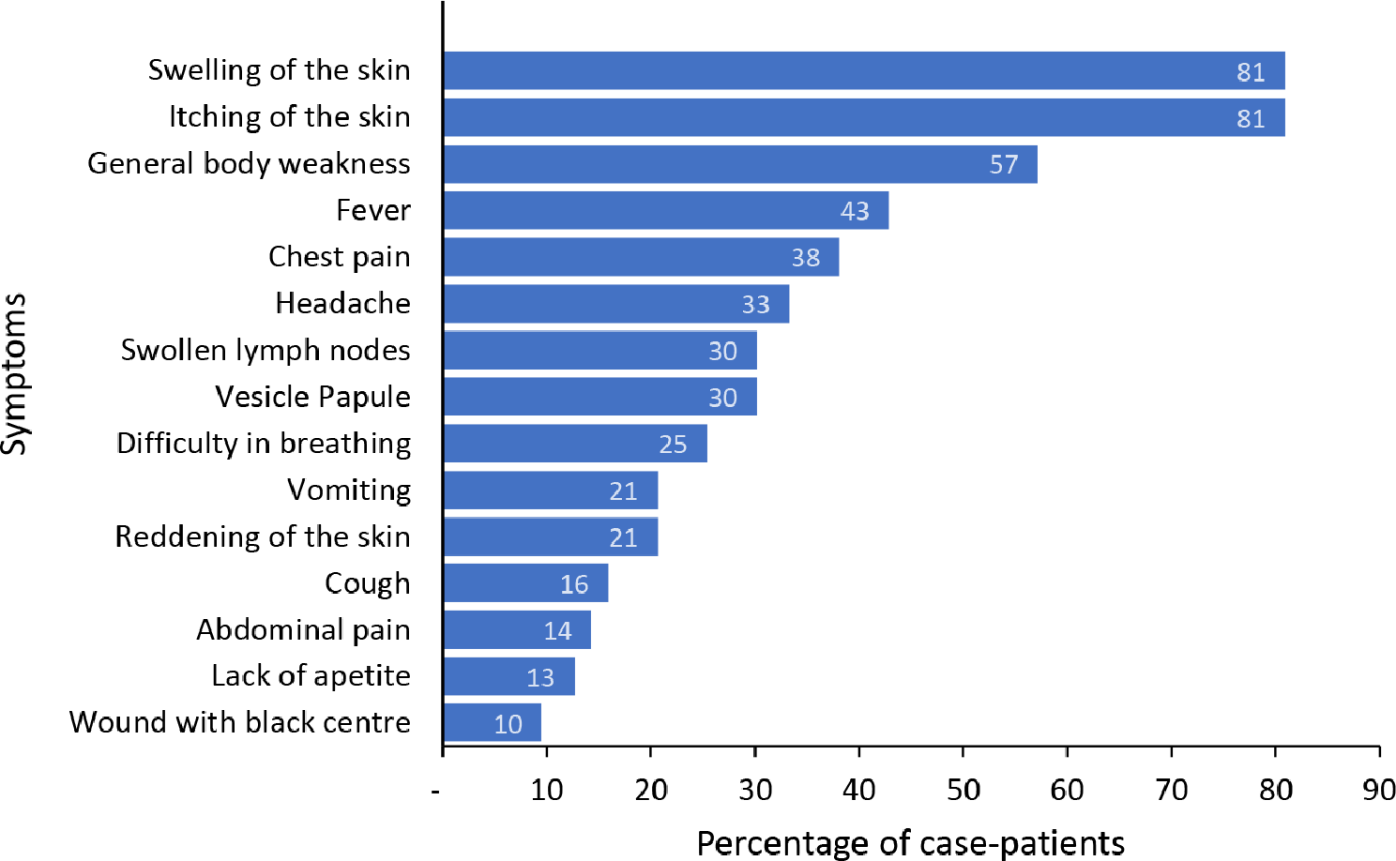
Distribution of symptoms among case-patients during an anthrax outbreak, Kyotera District, Uganda, June–December, 2023

We line listed 63 anthrax case-patients, with 17 confirmed and 46 suspected. 76% (*n* = 48) were males, 12 case-patients died, with an overall attack rate of 3.1/1,000 population and case fatality rate of 19%. Of the 63 case-patients, 55 were cutaneous and 8 gastrointestinal, with a mean age of 42 years (interquartile range: 13–75 years). Males were more affected (AR: 4.5/1,000) than females (AR: 1.5/1,000), age-group 50–69 was most affected (AR = 14/1,000). Swelling of the skin (81%), itching of the skin (81%), and general body weakness (57%) were the most common sign and symptom of illness among case-patients (Fig. 2). Only 29% of the case-patients sought care from Health facilities.

**Fig. 3:**
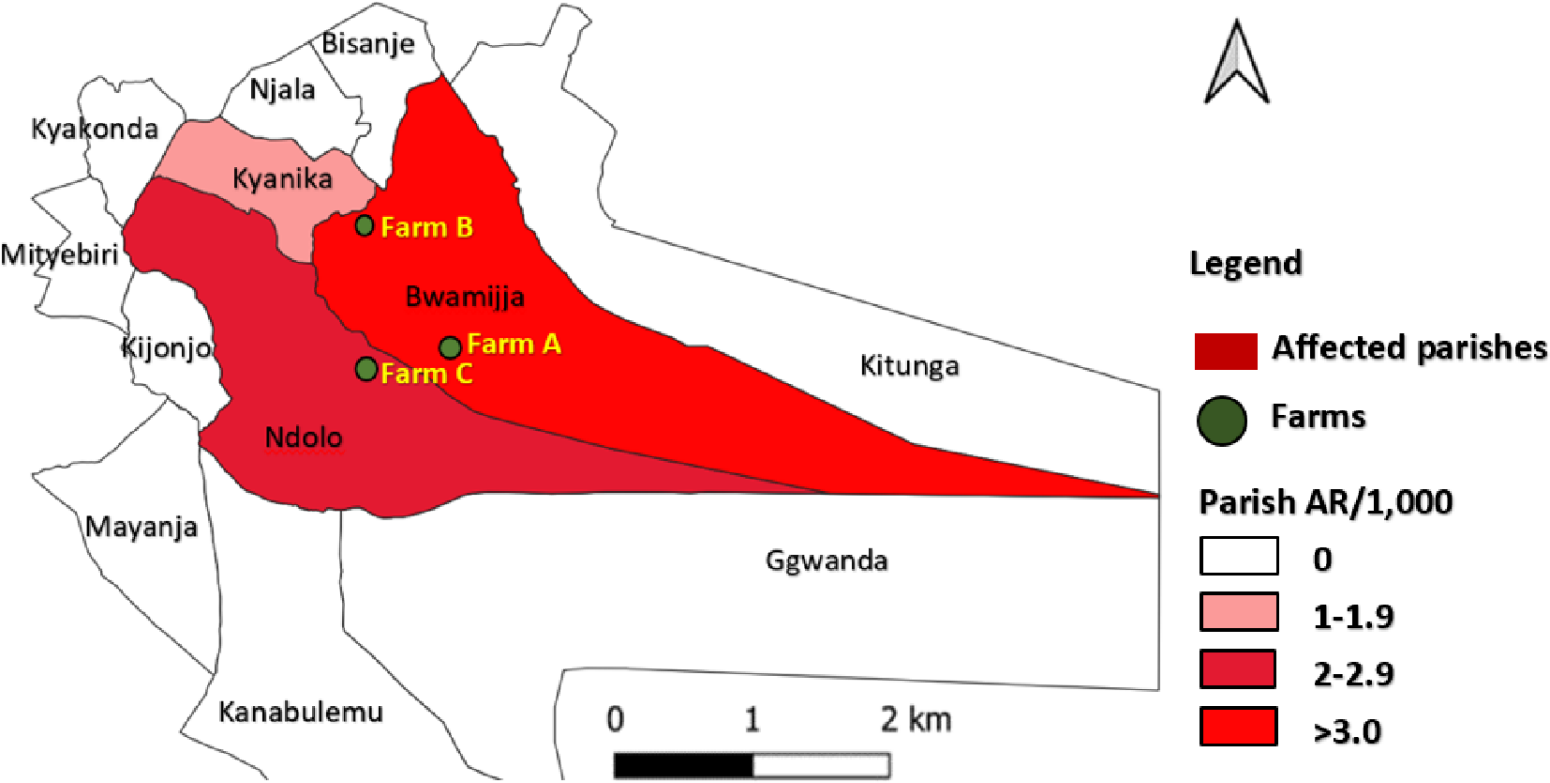
Location of the affected parishes and farms, Kabira Sub-county, during an anthrax outbreak, Kyotera District, June–December, 2023

Bwamijja parish was the most affected (AR: 5.3/1,000) and hosted two of the affected farms, followed by Ndolo (AR: 2.6/1,000) which hosted one affected farm while Kyanika was the least affected (AR: 1.4/1,000) (Fig. 3).

**Fig. 4:**
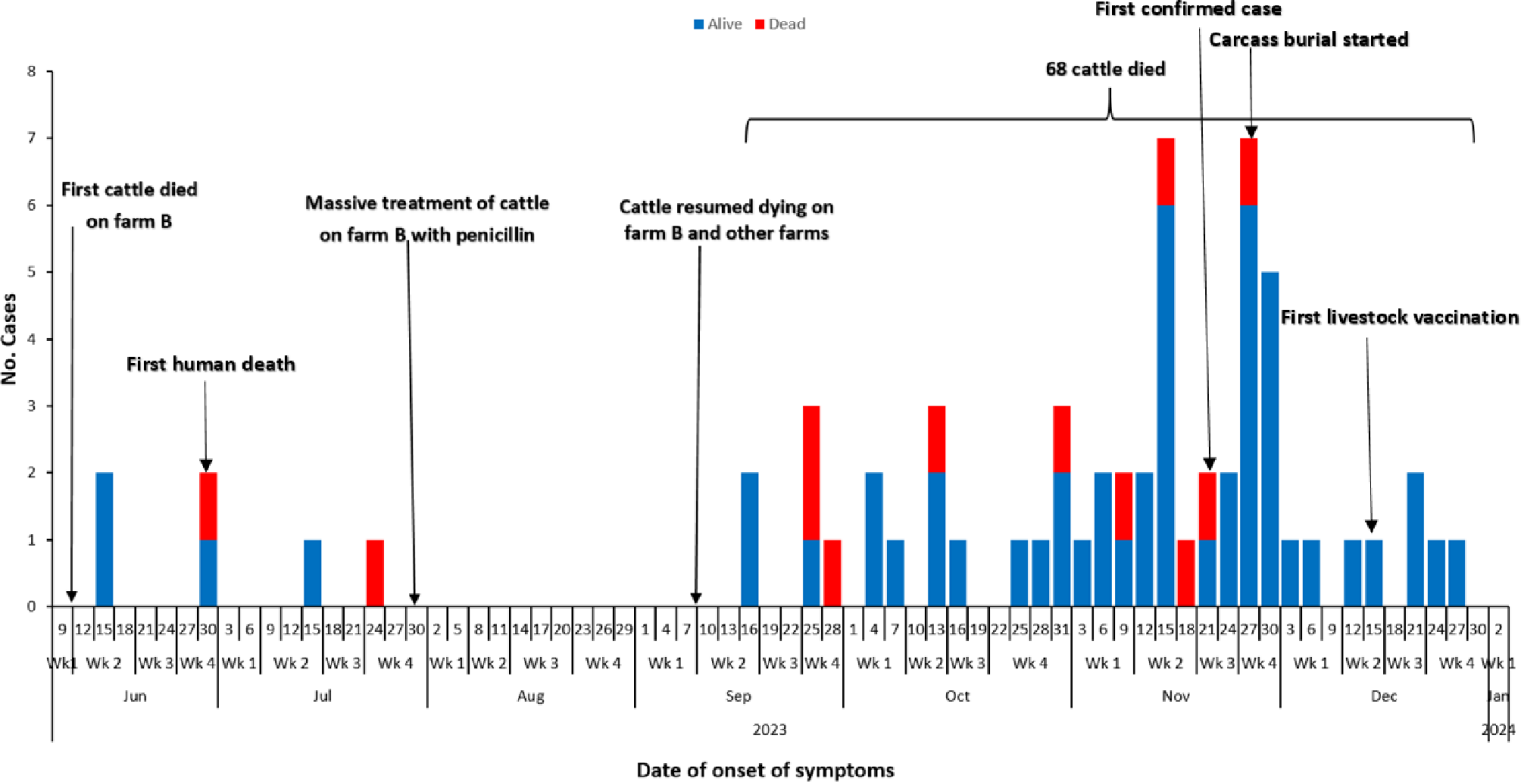
Distribution of anthrax cases by date of symptom onset, Kyotera District, Uganda, June–December, 2023

Following the sudden death and slaughtering of an animal on Farm B on June 10, 2023, case-patients started to appear from June 15, 2023 (Fig. 4). There was no case-patient recorded from July 27, 2023 up to September 13, 2023. This coincided with no animal deaths reported following a massive treatment of cattle on Farm B with penicillin therapy. Case-patients reappeared starting September 16, 2023 1 week after cattle had resumed dying on farm B and two other farms. The number of case-patients rapidly increased and peaked in November, 2023. This epidemic curve suggests a multiple-source outbreak. Overall, it took the district six months to initiate control measures to curb this outbreak.

### Laboratory investigation findings

Of the 57 tested samples,17 (30%) returned PCR positive for *Bacillus anthracis*.

### Environmental assessment findings

There were 68 suspected cases of animal anthrax that died on three different farms (A, B, & C) located in Kidda, Kyamayembe, and Kifambi villages in Bwamiija and Ndolo parishes respectively in Kabira sub-county. These three farms share one stream of drinking water with two of them (Farm A and C) having a common point of drinking water. The first animal died on farm B on June 10, 2023, and the dead animal was slaughtered by meat dealers who sold it to the surrounding community at the trading centers of Kyanika, Kyamayembe, and Kifambi at an average price of $1.28 per Kilogram. Farm B subsequently lost 22 cattle, and all of them were sold to the local community by the meat dealers. According to the farm manager, the deaths of cattle on farm coincided with the heavy rains. An organized network of people dealing in cheap meat from cattle that died suddenly was discovered in Kabira Sub-county, Kyotera District. We also identified an area next to dead meat stall where meat was prepared very fast for the slaughtering teams and people drinking in the next bar. Boiled meat termed ‘Huta ko’ was sold cheaply to people such that it’s affordable for everyone.

### Hypothesis generation interview findings

Based on the 63 hypothesis generating interviews, 79% of the respondents indicated they had consumed meat from cattle that had died suddenly, 63% had contact with dead livestock, wildlife or their body fluids, while 33% had participated in slaughtering or handling of animal products. We hypothesized that consuming and handling meat from cattle that died suddenly were associated with the December 2023 anthrax outbreak in Kyotera District.

### Case-control study findings

At bivariate analysis level, consuming meat and getting in contact with meat or other products from cattle that had died suddenly was associated with anthrax infection.

Following adjustment for age and sex, consuming meat from an animal that had died suddenly (aOR = 6.19; 95% CI: 2.76–13.9) and contact with meat or other products from cattle that had died suddenly (aOR = 2.52; 95% CI: 1.08–5.91) were the associated with this anthrax outbreak (Table 1).

**Table 1:** Distribution of exposure status among cases and controls during an anthrax outbreak: Kyotera district, Uganda, June–December, 2023. Reference: no contact with infected meat

Variables	Cases, *n* = 50(%)	Controls, *n* = 200(%)	Crude ORs (95% CI)	*P*-value	Adjusted ORs (95% CI)
Consumed dead cattle meat	37(74)	41(21)	11 (5.4–23)	˂0.001	6.2 (2.8–14)
Contact with meat or other products from dead cattle	31(62)	36(18)	7.4 (3.8–15)	˂0.001	2.5 (1.1–5.9)
Presence of hides in the household	6(12)	7(3.5)	1.3 (0.2–2.5)	0.02	0.8 (0.7–2.2)
Meat traders	5(8)	1(0.5)	2.9 (0.6–5.1)	0.01	2.6 (0.2-5.0)
Owned animals	27(54)	83(42)	0.5 (0.1–1.1)	0.11	
Cattle keeper	2(4)	14(7)	0.6 (-2.1-0.9)	0.2	

ORs: Odds Ratios; CI: Confidence Interval

Compared to persons that did not eat or have contact with cattle that died suddenly, the odds of acquiring anthrax were highest among persons who ate and had contact with cattle that died suddenly (OR = 20.9, 95% CI: 8.8–49.8 ), followed by persons that did ate meat but had no contact (OR = 5.81, 95% CI:2.12–15.9), with the lowest being among persons that had contact but did not eat meat from cattle that died suddenly (OR = 2.51, 95% CI:0.63-10.0)(Table 2).

**Table 2:** Distribution of exposure status among cases and controls during an anthrax outbreak: Kyotera district, Uganda, June–December, 2023. Reference: no contact with infected meat

Ate meat	Touched meat	ORs	*p*-value	95% CI
Ate meat	No contact	5.8	0.001	2.1–16
Didn’t eat	Contact with meat	2.5	0.194	0.6–10
Ate meat	Contact with meat	21	˂0.001	8.8–50

ORs: Odds Ratios; CI: Confidence Interval

## Discussion

The anthrax outbreak in Kabira sub-county, Kyotera District affected cattle and caused transmission to humans. Most of the cases of human anthrax were cutaneous, and the case fatality rate was higher than that reported in previous studies. Males were more affected than women, with the majority case-patients being managed by traditional healers. The outbreak was prolonged, and it took time to initiate control measures.

Contact with animal products and consumption of meat from cattle that suddenly died were the risk factors for this outbreak in Kyotera District. Skin itching and swelling were the most common symptoms, and were found on the hands than other body parts, a finding that was consistent with other studies [13, 14]. Since hands are used for handling meat, they are at higher risk of developing abrasions, bruises, and cuts, thereby creating a route of entry for the anthrax spores [18]. The study finding of cutaneous being more than gastrointestinal anthrax cases was in agreement with a study by Chakraborty A. et al. (2012) on anthrax outbreaks in Bangladesh which indicated that fewer gastrointestinal cases were due to cooking meat for a long period [15]. The process of cooking destroys the Bacillus anthracis thus reducing the virulence and chances of causing the disease. Additionally, the diagnosis of cutaneous anthrax can be made by considering the patient’s history and observing characteristic skin lesions. However, diagnosing gastrointestinal anthrax is more challenging given that its presentation is sometimes mild and similar to many other conditions like food poisoning, acute abdomen, and hemorrhagic gastroenteritis, thus the likelihood of missing them was high [3].

Males were more affected than females in this outbreak. In Uganda, men typically handle tasks such as slaughtering, skinning, and carrying animal parts, as well as roasting meat. This greater involvement in activities involving infected and dead animals increases their risk of cutaneous anthrax infection. Similar outbreaks have shown that men are more commonly affected than women due to these practices [9, 19–21].

The case fatality rate of 19% was higher than that reported in previous studies, this finding was contrary to other studies that had recorded low fatality cases in anthrax outbreaks [6, 7]. This was probably due to failure or delay to seek appropriate care especially during the initial stage of the outbreak as many people believed that witchcraft was the cause of the disease and thought care from traditional healers in shrines [22].

Eating and handling meat from cattle that died suddenly were found to be risk factors for contracting anthrax. These findings are consistent with previous studies of anthrax outbreaks in Uganda that found associations of anthrax to handling and eating of meat from cattle that died suddenly before slaughter [23, 24]. Since contact processes such as skinning and slaughtering increase the likelihood of developing cuts and abrasions, they can create pathways for spores to enter sub-dermal tissue. This study explored other factors that could influence risk levels such as handwashing and food preparation processes, and were found non-significant.

The environmental assessment showed some risk factors linked to animals contracting anthrax. The outbreak happened at the beginning of the rainy season, when there’s usually insufficient grazing grass, leading to limited grazing land and pastures. This shortage of grass increases the risk of grazing cattle ingesting anthrax bacilli due to overgrazing [25, 26]. Anthrax spores can survive for a long period when the soil conditions are favorable [27]. This outbreak could potentially have had environmental influences and we suggest future studies incorporating climate data.

A noteworthy finding is that the district took six months to implement outbreak control measures due to lack of an emergency response plan and insufficient personal protective equipment. Despite this delay, the outbreak was controlled within a month of confirming the first human case.The outbreak was curbed, as soon as the district started to institute outbreak control measures that included the recommended approach to bury animal carcasses deep down in the ground. The extensive health education and awareness campaigns conducted may have also played a significant role in curbing the outbreak [28, 29]. Studies have shown that increased community awareness and knowledge about anthrax in terms of mode of transmission, signs, symptoms, and preventive measures can reduce exposure to risk factors [30]. Strengthening inter-departmental coordination, enhancing active surveillance with daily reporting, and improving training for medical and veterinary personnel could mitigate future anthrax outbreaks in this setting [31].

Overall, the traditional practices of eating, selling, and distributing carcass meat common among these communities increase the risk of anthrax. It’s crucial to improve public health education, focusing on people involved in raising livestock, slaughtering, processing, cooking, and consuming meat [20]. The main message should stress the importance of avoiding any contact with, killing, consuming, buying, or selling meat from dead animals. Enforcement of stringent regulations could effectively discourage the practice of slaughtering dead animals. Raising awareness among the public and relevant personnel is crucial to promote responsibility in preventing anthrax. This will require concerted efforts from all departments, including the implementation of various behavior change models and information-education-communication (IEC) activities. Although some livestock vaccination against anthrax in Uganda is already in place, its coverage is currently insufficient since in most cases its individual livestock owners who carter for the vaccination costs inform procuring vaccines, transportation and allowances for veterinary officers who administer the vaccines. We recommend achieving maximum coverage of routine annual cattle vaccinations against anthrax. To reach effective herd immunity, at least 80% of the cattle in an area should be vaccinated [32]. Therefore, the Ministry of Agriculture, Animal Industry and Fisheries (MAAIF) should plan and support livestock owners with subsidized vaccination programs to achieve herd immunity. Although inter-departmental coordination exists in districts within Uganda, it needs further strengthening to ensure prompt responses to reports of animal deaths in specific locations. Improved active surveillance with daily reporting from the veterinary department is required to enable early detection, control, and prevention of outbreaks. Collaboration among the district health office, district veterinary office, and district forest office is essential for reporting suspected anthrax cases.

### Study limitations

Some cases may not have been line listed during case-finding given that many people were managed in secret shrines by traditional healers and we could not be allowed to access them. This likely led to an underestimation of the magnitude of the outbreak. We were also unable to obtain samples from people who died before the team had reached the field, although the team was able to interview the caretakers and review medical records to determine whether they met the case definition. The collateral history from some caretakers might have had recall issues on the sequence of events given that they did not experience the signs and symptoms themselves. Additionally, some anthrax cases only develop mild symptoms and may not know that they have anthrax, so there was a likelihood of missing them out during case finding; this may have potentially underestimated the magnitude of this outbreak. To minimize the risk of overlooking case-patients due to the lengthy time frame, each interviewee was asked about others who exhibited similar symptoms. It is possible that some cases were not true instances of anthrax, which could lead to an overestimation. However, all case-patients presented with skin lesions that progressed to black eschar, which is a hallmark sign of cutaneous anthrax. Therefore, they were classified as anthrax cases. The case definition specifically included skin lesions and black eschar, ensuring that case-patients with cutaneous anthrax were accurately identified. In order to rule out other skin and gastrointestinal illness, each interviewee was asked about the sequence of symptoms and case-patients reported symptoms after contact with livestock that had died suddenly, establishing a possible link.

## Conclusion

Eating and handling meat from cattle that died suddenly was associated with the anthrax outbreak in Kabira Sub-county, Kyotera District. The outbreak could potentially have been influenced by environmental factors especially with rainfall increasing anthrax spore exposure. We recommended inspection of all cattle prior to slaughter and vaccination of livestock against *B. anthracis.* We also recommended continued risk communication and community engagement sessions regarding anthrax throughout the District, as well as culturally sensitive strategies to encourage early healthcare-seeking.

### Public health actions

Following our recommendations, the task force immediately announced an animal quarantine for 14 days. Additionally, we sensitized resistant affected communities to create anthrax awareness and enable them cooperate with the response teams. We also supported farm managers to properly dispose off animal carcass whenever an animal died on the farm.

## Data Availability

The datasets upon which our findings are based belong to the Uganda Public Health Fellowship Program. For confidentiality reasons, the datasets are not publicly available. However, the datasets can be availed upon reasonable request from the corresponding author with permission from the Uganda Public Health Fellowship Program.

## Abbreviations

AOR: Adjusted Odds Ratio
CI: Confidence Interval
CPHL: Central Public Health Laboratories
LC: Local council
MoH: Ministry of Health
NADDEC: National Animal Disease Diagnostics and Epidemiology Centre
OR: Odds Ratio
PCR: Polymerase Chain Reaction
UVRI: Uganda Virus Research Institute
VHT: Village Health Team
